# Benefits of Categorizing Noncovalent Bonds Based on Hydrogen, Halogen, Chalcogen, and Pnictogen Bonds

**DOI:** 10.1002/anie.6901593

**Published:** 2026-02-17

**Authors:** Andrea Pizzi, Giancarlo Terraneo, Cristina Lo Iacono, Roberta Beccaria, Arun Dhaka, Giuseppe Resnati

**Affiliations:** ^1^ NFMLab, Department of Chemistry, Materials, Chemical Engineering “Giulio Natta” Politecnico di Milano Milano Italy

**Keywords:** chalcogen bond, halogen bond, nomenclature, pnictogen bond, weak interactions

## Abstract

Recent IUPAC recommendations define the halogen bond, the chalcogen bond, and the pnictogen bond as the interactions between nucleophilic sites and electrophilic elements of groups 17, 16, and 15 of the periodic table. In a recent paper Robin Taylor states that, the use of these three terms should be “deprecated” and he raises concern about the proposal that we made in a scientific perspective on this journal to use these three terms, along with the hydrogen bond one, as the core of a taxonomy of chemical interactions, wherein names refer to the electrophile group/atom. Here, we show how Taylor's position conflicts not only with the authoritative position of IUPAC, but also with the common practices of the chemists’ community. Our proposed naming scheme which refers to the electrophile group/atom far from concealing key information, as assumed by Taylor, enables for a precise language whose terminological richness is instrumental in communication efficiency. Our naming scheme presents the advantage to be invariant with respect to the evolving understanding of the nature of chemical interactions and to be tailored to automated database mining, ontology construction, and AI‐driven structure–property prediction.

## Graphical abstract

A terminology for electrophile‐nucleophile interactions based on names that refer to the electrophile group/atom may offer the advantage to be descriptive, consistent, complete, systematic, clear, and, most important, invariant. Likely, a terminology that employs only the terms σ‐hole bond, π‐hole bond, and p‐hole bond may not.

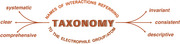

## 1 Introduction

According to the IUPAC definition, the halogen bond (HaB) is the net attractive interaction between an electrophilic region associated with a halogen atom in a molecular entity and a nucleophilic region in another, or the same, molecular entity [1]. The HaB is a relatively new entry on the palette of the noncovalent bondings, but it has already become a valuable tool in all fields wherein recognition and self‐assembly play a role [2]. Many other interactions wherein the electrophile is an element of the *p* or *d* blocks of the periodic table are commonly named after the group of the periodic table to which the electrophile belongs. The chalcogen bond (ChB) [3] and pnictogen bond (PnB) [4] are the IUPAC defined terms for the interactions wherein the electrophile is an element of the groups 16 and 15. The tetrel bond (TtB) [5], noble gas bond (NgB) [6], spodium bond (SpB) [7], regium bond (RiB) [8], matere bond (MaB) [9], and titan bond (TnB) [10], the last entry in the group, are names used to designate bondings wherein the electrophile is of the groups 14, 18, 12, 11, 7, and 4. If greater specificity is pursued, interaction have also been named after the element acting as the electrophile; for instance, the chlorine bond and the bromine bond are the HaBs wherein the electrophile is chlorine and bromine and the sulfur bond and the selenium bond are the ChBs wherein the electrophile is sulfur and selenium.

In 2014 we advocated the wide‐ranging potential of naming electrophile‐nucleophile interactions by referring to the electrophile group/atom [11]. Few months ago, in a scientific perspective on this journal, we proposed this naming scheme as the core of a taxonomy of chemical interactions, this core offering the advantages to be descriptive, systematic, consistent, and most important invariant [12].

In a correspondence, once again on this journal, R. Taylor raised concerns about our proposals in the scientific perspective [13]. A major objection to the naming of interactions “after the periodic table group to which the electrophile belongs or, if greater specificity is required, after the element type of the electrophile” is that this protocol “conceals the strong underlying similarity of the differently named interactions”. Taylor goes on stating that “the obvious alternative is to use the terms that reflect the physical natures of these interactions: σ‐hole bond, π‐hole bond, and possibly p‐hole bond”. Here we reply to Taylor's correspondence and shown how Taylor's concerns and considerations:—are unfounded and based on misunderstandings;—ignores a key part of the message of our scientific perspective, for example, the relationship, namely complementarity, between the terms based on the electrophile group/atom and other terms, the hole‐based ones included;—leave unrefuted the soundness of our proposal, that is a taxonomy based on terms which refer to the electrophile group/atom. In the next section we restate that, our contribution towards a “common understanding of commonly used language” [14] consists in taxonomizing, that is, in complementing, without any possibility of concealing. The section after the next one presents considerations that have not been given in the scientific perspective [12], and that further prove the value and the advantages of our proposed naming protocol.

### 1.1 Alleged Concerns on Interaction Names That Refer to the Electrophile Group/Atom

**FIGURE 1:**
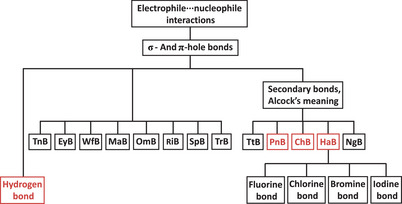
Tree notation of a chemical interactions taxonomy based on a systematic reference to the electrophile. In red the IUPAC defined terms. Other terms could be added at different levels of generality (e.g., carbon bond and selenium bond at the lowest level of generality where the fluorine bond and other subsets of the HaB are reported). The image above is the same as the image of Figure 33 of our Scientific Perspective (ref. [12]) except for the box of the TnB.

“Simplicity, greater clarity (our note: with respect to the terminology based on electrophile group/atom) and lack of ambiguity” are the benefits claimed by Taylor for the σ‐, π‐, and p‐hole bonds (henceforth σ/π/p‐hole bonds) terms in the abstract of his correspondence. Further on in the text of the paper he acknowledges that, being able to go from general to specific is essential and as an example of this ability for the σ/π/p‐hole bonds terms he mentions the triad σ‐hole bond, halogen σ‐hole bond, and bromine σ‐hole bond. Elsewhere in the correspondence Taylor proposes to use other compound terms wherein words referring to the eletrophile group/atom are employed as prefixes or postfixes of σ/π/p‐hole bonds terms, for example, σ‐chalcogen bond or π‐hole tetrel bond, and carbon π‐hole bond. These compound terms are a breeding of the σ/π/p‐hole bonds terminology and of the one referring to the electrophile group/atom. Figure 1, represents after the tree notation our proposed taxonomy of chemical interactions based on a systematic reference to the electrophile group/atom. Figure 1 is an updated version of Figure 33 of our scientific perspective [12], the only difference being the box of the titan bond (TnB) [10], an interaction which has been described after the publication of the scientific perspective. Interestingly, the triad mentioned above is the transposition into words of a part of Figure 1, that is of Figure 33 of our scientific perspective. Rather than disproving the usefulness of terms which refer to the electrophile group/atom, Taylor's compound terms confirm that such referring is essential whenever the going from general to specific is pursued.

Dozens of such compound terms are required to describe the interactions in the diversity of existing molecular entities and the “simplicity” claimed by Taylor for the terminology that uses the σ/π/p‐hole bonds names are thus lost whenever greater descriptiveness is pursued, that is according to Taylor's wording, when “greater clarity and lack of ambiguity” are the targets.

A Taylor's criticism to the naming protocol that refers to the electrophile group/atom is that it causes a detrimental proliferation of term. But since being able to go from general to specific is essential, in his proposed substitute terminology, he ends up with a proliferation analogous to that imputed to the naming scheme that we propose in the scientific perspective. The soundness of our proposal to use a taxonomy based on terms referring to electrophile group/atom is confirmed rather than refuted. Indeed, the taxonomy that we propose, far from introducing unnecessary complexity, seeks to organize existing complexity [15]. It provides a language that integrates empirical and theoretical understanding and can be equally useful to scientist involved in quite different fields (crystallography, spectroscopy, computational, and supramolecular chemistry). Our machine‐friendly taxonomic nomenclature supports database mining, ontology construction, and AI‐driven structure–property prediction, areas where “simplicity” without semantic precision engenders problems.

**FIGURE 2:**
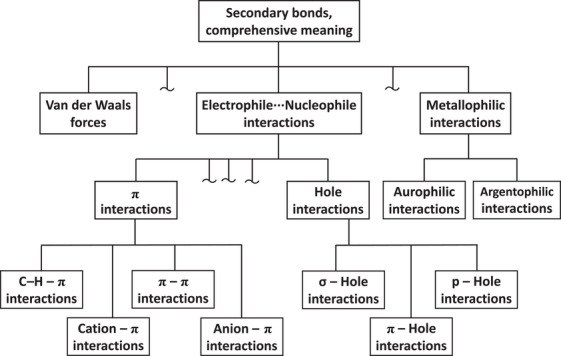
Tree notation of the hierarchical organization of some terms used to designate bondings. The image of this figure is the same as the image of Figure 27 of our scientific perspective (ref. [12]).

The multiplicity of names used to designate interactions when referring to the electrophile group/atom is a plus of this terminology. When the reference is given to the electrophilic atom, the respective term allows for acknowledging and declaring the highly specific features of the interactions formed by one element of a group versus interactions formed by other elements of the same group. When the reference is given to the group of the electrophilic atom, the respective term acknowledges and declares the less specific commonalities of the interactions formed by the elements of that group versus those formed by the elements of other groups. As considered in detail in the text of our scientific perspective and represented in its figures, when the discussion on an interaction aims at focussing on even less specific and more general commonalities, other terms are available and should be used, the most convenient term depending on the generality of the considered commonalities. For instance, the terms σ‐hole bond, π‐hole bond, *p*‐hole bond, or hole‐bond, or secondary bond can be used if the considered commonalities have smaller, or mid, or greater generality (Figure 2). This is a unique advantage of our proposed hierarchical terminology for chemical interactions and resulting taxonomy whose different terms refer to sets and subsets of interactions sharing commonalities with different levels of generality.

In the introduction of his correspondence, Taylor defines a σ‐hole as the “region of depleted electron density on the opposite side of an atom to a σ‐covalent bond” and a σ‐hole bond as the directional interaction between a σ‐hole and a nucleophile. A π‐hole and a π‐hole bond are analogously defined in relation to an atom forming a π‐bond and interacting with a nucleophile. In our scientific perspective we considered some weaknesses of interaction names that refer to the forces responsible for interactions formation, of the terms σ/π/p‐hole bonds included. In countering our considerations Taylor states that “it is the real interaction that needs to be named” and continues saying that “consideration of the isolated starting molecule is irrelevant”. This sentence implies that the σ‐holes and the π‐holes (defined by Taylor for an atom in a molecular entity with no reference to surrounding/interacting molecular entities) are irrelevant. It seems there is something inappropriate in his phrasing since if the σ‐holes and the π‐holes are irrelevant to the naming of interactions, the terms σ/π/p‐hole bonds cannot be the “obvious alternative” to the terms referring to the group/atom as Taylor proposes.

According to the IUPAC definitions, the chalcogen bond [3] is the “net attractive interaction between an electrophilic region associated with a chalcogen atom in a molecular entity and a nucleophilic region in another, or the same, molecular entity” and the pnictogen bond [4] is the “weak attractive interaction between an electrophilic region on a pnictogen atom in a molecular entity (wherein the pnictogen is involved in other stronger bonds) and a nucleophilic region in another, or the same, molecular entity”. Taylor states that, these definitions are “nearly identical”. The wordings “net attractive interaction” and “weak attractive interaction” patently refer to different interactions, not to nearly identical interactions. It thus seems that Taylor misjudges two of the IUPAC definitions of interactions that are at the core of the naming scheme which we propose in our correspondence. The two cases described above exemplify that, the consistency of Taylor's paper is weak from both the logic and the semantic point of view even on very fundamental aspects of the naming scheme that he suggests as well as of the scheme that we proposed and he counters.

Taylor writes that in our scientific perspective we “give examples of atoms that do not have positive σ/π‐holes when the molecules to which they belong are isolated, but develop them when polarized by the approach of one or more other molecules and consequently form short contacts to nucleophiles”. He then continues quoting us: “In their words: «It is obviously hard to name these short contacts σ/π‐hole bonds (these terms asking by definition for the presence of σ/π‐holes)»” The sentence quoted by Taylor from our manuscript (i.e., that in angle brackets «») follows, in our paper, the discussion of cases wherein an electrophilic and a nucleophilic site are in the same molecule, interact intramolecularly, and form a short contact locking the molecule in a conformation that buries the hole inside the molecule. Clearly, our text quoted by Taylor refers, in our scientific perspective, to molecules which have no hole on their surface, either when isolated or when interacting with surrounding molecules. Our text quoted by Taylor does not refer to molecular entities which do not have holes as isolated species and develop holes when polarized by surrounding molecular entities, as indicated by his narrative. In our scientific perspective, these last species are discussed in a completely different context and several paragraph further on.

This is an example of a practice that Taylor uses repeatedly in his correspondence: He picks up issues and statements that, in our text, are distant from each other and are not logically linked with each other. He puts them in his correspondence side by side, thus casting doubts on our reasonings and attributing to us, sometimes implicitly, sometimes explicitly, conclusions that are not present in our text.

Another such case is where Taylor quotes our statement «the understanding of the halogen bond was perfected» to give the message that our opinion and IUPAC definition of the HaB match with his opinion that the HaB nature is “entirely coulombic”. Indeed, when in our scientific perspective we state, «the understanding of the halogen bond was perfected», we refer to the pathway which ended up with the IUPAC definition of the halogen bond where it is stated “the forces involved in the formation of the halogen bond are primarily electrostatic, but polarization, charge transfer, and dispersion contributions all play an important role”.

In our scientific perspective it is clearly stated that the terminology using names which refer to one of the forces contributing to the interaction, is convenient when the reference is made to “the prevailing force” that allows for the interaction formation or when the purpose is to draw attention to this force component, while in other cases this naming protocol is “misleading as the attention is drawn to a minor aspect of the interactions rather than to the major one(s)”. This position aligns with a widely shared opinion (see onward) that the HaB and similar hole interactions are, as formulated by S. Kozuch, “An amalgamation of several key effects: electrostatic, polarization, dispersion, and charge‐transfer. The weight of each one of these components depends on the type of moieties in the adduct, on the bond distance, on the method used to quantify the effects, and some would even say that it also depends on the researcher's whim” [16]. A quite similar position was adopted (see above) by the IUPAC definitions of the HaB [1] and the PnB [4].

Some years ago, it was Taylor's position too: In 1996, in one of his papers discussing attractive interactions between electrophilic halogens and nucleophiles it was written: [17] “The attractive nature of the interaction is mainly due to electrostatic effects, but polarization, charge‐transfer, and dispersion contributions all play an important role in causing interpenetration of van der Waals volumes.” But it seems that Taylor changed his opinion on the attractive forces responsible for the formation of the HaB and analogous interactions formed by the elements of other groups, as in the correspondence he states that, “the forces on the nuclei in a molecule or molecular complex (and, by extension, intermolecular interaction energies) are entirely coulombic in nature”. Consistently, he now proposes that all the interactions under consideration should be named “σ‐hole bond, π‐hole bond, and possibly, p‐hole bond”, terms that refer specifically to the electrostatic force.

Interestingly, in 2024, Taylor published a paper wherein he was proposing a substitute terminology of the one which refers to the electrophile group/atom [18]. But that substitute terminology was different from the one proposed in the Correspondence. In 2024 he claimed that “the simplest alternative nomenclature” to that referring to the electrophile group/atom is “σ‐hole interaction, π‐hole interaction, and p‐hole interaction, as well as perhaps peri hypervalent bond and peri covalent bond”.

Many cases are available in the literature wherein the opinion on the forces allowing for the bondings formation has changed over time, typically when advances in existing techniques or introduction of new techniques open the way to deeper insights. A nomenclature of interactions that, refers to the forces responsible for their formation, can accordingly change over time, and, as sketched above and discussed in the detail in our scientific perspective, it did change for the HaB and analogous interactions. The very changes of Taylor's statements may be considered a case in point. A deeper insight of the forces determining interactions formation is an advancement of science, a change of the interaction nomenclature is not as it complicates communication and information storage and retrieval. The elements forming an interaction do not change over time and an interaction nomenclature based on these elements, specifically the electrophilic ones, is invariant over time. This aspect has been presented in our scientific discussion, but it seems Taylor undervalues its relevance and we thus stress again the added value of our proposed naming scheme with respect to the σ/π/p‐hole bond nomenclature commended by Taylor. Importantly, the added value applies to both human and machine‐based protocols (e.g., machine learning, automated logic extraction, and reliable algorithm training) that are becoming more and more important.

The weakness of Taylor's proposed naming scheme and of the arguments supporting it goes far beyond the countering of our proposed taxonomy as it substitutes theoretical reductionism for chemical descriptiveness. In principle, it can be accepted that all bonding interactions are coulombic, but the limiting to this viewpoint affords no chemical insight. Chemists typically classify bonds by key features (ionic, covalent, …) despite all being Coulombic in origin. With a daring generalization, it can be stated that to limit the naming differentiation on the basis of universal Coulombic origin would collapse the entire understanding of chemical interactions into electrostatics. The descriptive value of our taxonomy results precisely from it reflecting the diversity of bonding phenomena, leaving to the field of theoretical chemistry the discussion of the unity of underlying physics.

### 1.2 Value of a Taxonomy of Interactions Based on the Electrophile Group/Atom

In this section, we tackle three additional issues to confirm the value of the approach proposed in our scientific perspective. The first is a chemical issue, the second one is drawn from linguistics, and the third one relates to consensus.

Probably, the organic frameworks (OFs) whose crystalline or amorphous forms show porosity are one of the most exemplary cases wherein the hierarchical and taxonomic classification are usefully employed in chemistry. The assignment of 2025 Nobel prize to S. Kitagawa, R. Robson, and O. Yaghi recognized the paramount novelty and importance of these systems whose applications span fields as diverse as harvesting water from desert air, capturing carbon dioxide and toxic gases, and catalyzing chemical reactions.

The varied set of the OFs has been divided into subsets as a function of the nature of the starting molecular entities or of the bonds assembling these entities into the final porous materials. The systems wherein metal nodes are connected to organic spacers by coordination bonds are named metal organic frameworks (MOFs) [19]. The systems wherein the component molecular entities are solely organic are named supramolecular organic frameworks (SOFs) [20] and this latter set has been divided in several subsets as a function of the bondings driving the assembly of the molecular entities into the final constructions. The covalent organic frameworks (COFs) [21] and the noncovalent organic frameworks (nCOFs) [22] are the systems assembled via covalent and noncovalent bonds, respectively. The hydrogen bonded organic frameworks (HOFs) [23] and the halogen bonded organic frameworks (XOFs) [24] are subsets of the nCOFs, specifically the systems assembled via hydrogen and halogen bonds. The chemical and functional properties of these systems, for example their stability and solvent processability, obviously differ from each other. To tune these properties, systems have also been described wherein two interactions contribute to the starting modules self‐assembly, for example this is the case for the halogen‐hydrogen bonded organic frameworks (XHOFs) [25].

As it is often the case for the relationship among different chemical terms [12], some other commonly used terms identify sets of porous systems which intersect with the sets considered above or which are their subsets. For instance, the set of the porous molecular crystals (PMCs) [26] is a subset of the OFs set and intersects with the HOFs and XOFs sets. The commonly used hierarchical categorization and taxonomic classification of systems as important and diversified as the porous materials supports the usefulness of categorizations and classifications of the chemical interactions.

Linguistic research performed on many different languages (more than two thousand) and text types (spanning legalese or religious texts and subtitles or talks in movies) revealed that greater text complexity [27] tends to co‐occur with shorter text length [28]. Obtained results suggest that this inverse relationship between complexity and length is a compensatory mechanism, which allows for increased complexity to be offset by increased efficiency. In other words, more complex languages offer the payback that fewer symbols are required to convey the same message. The trade‐off between complexity and efficiency which regulates how languages evolve “is shaped by the social environments in which languages are used, with larger communities tending to use more complex but more efficient languages” [29]. To learn a complex language is more demanding than to learn a simple one, but “once you've mastered it, a complex language might offer more options to express yourself” and it becomes easier to encode a given idea using fewer symbols. It has also been speculated that [29] “in large societies, institutionalized education might enable greater linguistic complexity by providing systematic and formalized language learning, which supports the acquisition and use of intricate linguistic structures. At the same time, the importance of written communication in larger societies may create pressure for shorter messages to reduce costs for production, storage, and transmission”.

All these linguistic considerations consistently support the proposal for the rich and diversified, that is, more complex, terminology fostered in our scientific perspective rather than the three‐terms, that is, less complex, terminology proposed by Taylor. A high education is common in the academic community and this allows all its members to master the numerous terms in use when referring to the electrophile group/atom. These numerous terms allow for more efficient information storage and data processing through the increasingly important machine‐based protocols.

The common use of a terminology is a key aspect of its usefulness and reveals a consensus on its sematic value, namely on its ability to bridge language, world, and mental concepts. On 30^th^ October 2025 a web of science—core collection search in the last five years for “halogen bond” as the topic afforded 4650 entries. A non‐minor fraction of these papers is published in highly impacting journals (for example, Science, Nature portfolio, J. Am. Chem. Soc., Angew. Chem. Int. Ed.) and 44 of them are “Highly Cited Papers” (HCPs) [30]. Smaller numbers of papers, but with analogously high qualitative level are using the terms “chalcogen bond” or “pnictogen bond” [31]. Clearly, many scientists with quite different expertise and among them several particularly eminent scientists [32], have considered it was convenient to use this terminology, sometimes even in the titles of their papers [33, 34, 35]. Reviewers and peers allowed some of these papers to be particularly impacting. This proves the consensus in the scientific community on the naming interactions by referring to the electrophile group/atom. Taylor has a radically different position, in the conclusion of his correspondence, he states that the use of the IUPAC approved terms halogen bond, chalcogen bond, and pnictogen bond should be “deprecated”. He acknowledges that, the approval of IUPAC definitions “involved multiple rounds of peer‐review in a process open to comment by the whole chemistry community”, but he does not consider that this ensures the value of these terms and he also ignores that their use by many scientists, some of them particularly eminent, is an acknowledgement of their value.

## 2 Conclusions

Considerations above confirm that differentiation in terminology strengthens, rather than weakens, chemical discourse. Our proposed taxonomy provides a periodic and scalable framework for noncovalent interactions, integrating established terms such as hydrogen, halogen, chalcogen, and pnictogen bonds while accommodating future discoveries. Far from multiplying terms arbitrarily, it offers a coherent structure that connects them under a unified logic based on the identity of the electrophilic centre. Far from unduly inflating language, our naming scheme provides an enduring structure that reflects the diversity and depth of modern noncovalent bonding concepts.

**FIGURE 3:**
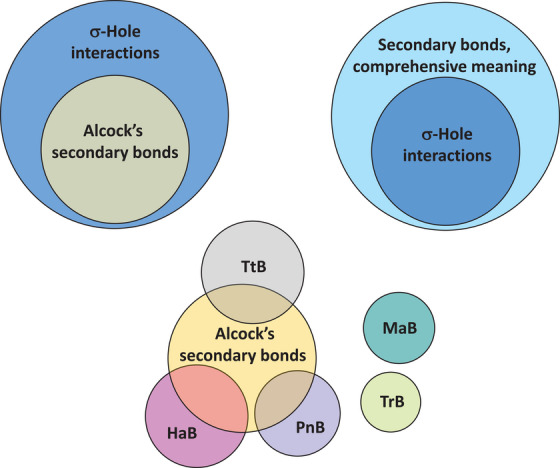
Venn notation of the relationship between different sets of interactions. Top: the secondary bonds according to Alcock's meaning are a subset of the σ‐hole interactions which, in their turn, are a subset of the secondary bonds when the term is used according to the comprehensive meaning. Bottom: the tetrel bond (TtB), the pnictogen bond (PnB), and the halogen bond (HaB) are sets of interactions intersecting with the set of the secondary bonds according to Alcock's meaning while the matere bond (MaB) and the triel bond (TrB) are disjoint sets. The image of this figure is the same as the image of Figure 1 of our scientific perspective (ref. [12]).

**FIGURE 4:**
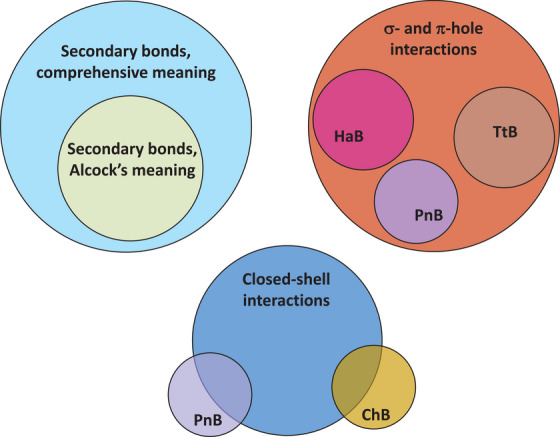
Representation according to the Venn notation showing how: The Alcock's secondary bonds are a subset of the secondary bonds according to the comprehensive meaning (top, left); HaB, ChB, and PnB are subsets of the σ‐ and π‐hole bonds set (top, right); the ChB and PnB intersect with the closed‐shell interactions (bottom) (e.g., SF_4_, Martin reagent, and SbCl_5_^2–^ have more than eight valence electrons and form ChBs and PnBs, respectively). The image of this figure is the same as the image of Figure 28 of our scientific perspective (ref. [12]).

Taylor neglects our pages long presentation of the hierarchical categorization of the terms σ/π/p‐hole bonds relative to the terms referring to the electrophile group/atom and he completely ignores our representation of their logical relationships after the Venn notation in Figure 1 and 28 of our scientific perspective [12] (reproduced in Figures 3 and 4 of this manuscript) and after the tree notation in Figures 27 and 33 of our scientific perspective [12] (reproduced here in, Figures 1 and 2, respectively). Our proposed taxonomy of chemical interactions (Figure 1) includes both hole‐based terms and electrophile group/atom‐based ones, namely it acknowledges and declares their complementary semantic and heuristic value, as logically required by the different component terms of a taxonomy. Taylor raises concerns on our naming scheme which, refers to the electrophile group/atom, but at the same time he proposes compound names which are breeding the σ/π/p‐hole bonds terminology and the terminology referring to electrophile group/atom.

In our scientific perspective, documental proofs based on experimental and theoretical findings, not at all “based on irrelevant hypothetical interactions” as claimed by Taylor, corroborate the robust character (i.e., descriptive, comprehensive, systematic, consistent, and invariant character) of names of interactions referring to the electrophile group/atom with respect to names referring to other interaction features.

As stated in the concluding section of our scientific perspective, one of the aims of that paper is “to favor a discussion on the logic underlying the terms used to designate chemical interactions”. It is well‐established that the more basic, or innovative, an issue is, the more tortuous the path towards a scientifically robust consensus is; the hydrogen bond concept and term are a particularly pertinent example [36]. Constructive criticisms to the proposals made in our scientific perspective will help in reaching better terminologies for designating the weak bonds and the same holds for disputing which use crystal clear arguments. Proactive scientific debate is a highly valued practice for advancing science; contradictory rhetoric, prejudicial confront, and allegations are not. A rigorous and descriptive language helps scientific communication, a fluid and metamorphic language does not as it is more tailored to poetry than to science.

Structured differentiation not minimalism in chemical nomenclature is vital for clarity, communication, and progress. The history of the halogen bond illustrates this point vividly. The designation of the phenomenon with a distinct term transformed a set of scattered observations into a coherent, interdisciplinary field spanning supramolecular chemistry, drug design, catalysis, and materials chemistry. Naming the interaction did not complicate communication, it enabled it. Experimental chemists, structural biologists, pharmacologists often need names that immediately convey chemical context. Group and element specific nomenclature provides this, facilitating clear discussion of observed geometries and functional roles. Moreover, explicit, hierarchical naming supports machine‐readable databases, computational classification, and AI‐assisted analysis that are increasingly central to modern chemical research.

## Conflicts of Interest

The author declares no conflict of interest.

## Data Availability

The data that support the findings of this study are available from the corresponding author upon reasonable request.
